# Editorial: Cannabidiol: perspectives on drug-resistant epilepsy management

**DOI:** 10.3389/fphar.2026.1977379

**Published:** 2026-09-23

**Authors:** Jerónimo A. Auzmendi, Antonio Gil-Nagel, Dolores E. López García, Alberto Lazarowski

**Affiliations:** 1 Instituto de Neurociencias de Castilla y Leon, Salamanca, Spain; 2 School of Pharmacy and Biochemistry, Institute of Pharmacological Research (ININFA- UBA-CONICET), University of Buenos Aires, Buenos Aires, Argentina; 3 Director of Epilepsy Unit, Hospital Ruber Internacional, Madrid, Spain; 4 Instituto de Investigación Biomédica de Salamanca (IBSAL), Salamanca, Spain; 5 School of Pharmacy and Biochemistry, Institute for Research in Physiopathology and Clinical Biochemistry (INFIBIOC), Buenos Aires, Argentina

**Keywords:** cannabidiol, inflammation, molecular targets, personalized therapy, refractory epilepsy

This Research Topic aims to highlight advances in the clinical use of cannabidiol (CBD) in drug-resistant epilepsies and to explore the biological and molecular mechanisms of action emerging from experimental models. Consistent with the scope of the Drugs Outcomes Research and Policies section, the works gathered here are anchored in post-approval clinical outcomes—effectiveness, safety, monitoring and access—of pharmaceutical-grade, highly purified oral CBD (Epidiolex® in the US; Epidyolex® in the EU), the only CBD formulation supported by regulatory approval and by pivotal randomized controlled trials in Dravet syndrome, Lennox-Gastaut syndrome and tuberous sclerosis complex (Devinsky et al., 2017; Thiele et al., 2018; Thiele et al., 2021).

The two preclinical studies in this Research Topic are discussed as sources of mechanistic and translational insight that support the clinical evidence base, rather than as post-approval outcome data.

The work presented by Navarro not only summarizes the practical aspects of prescribing and administering highly purified CBD oral solutions in patients with drug-resistant epilepsy (DRE-operationally defined, per the ILAE consensus, as failure of adequate trials of two tolerated and appropriately chosen antiseizure medication schedules to achieve sustained seizure freedom; Kwan et al., 2010), but also addresses different dosing strategies and the profile of potential adverse drug reactions (ADRs). This narrative review emphasizes the different mechanisms of action of CBD in epilepsy, its pharmacokinetics and its pharmacological interactions with anti-seizure medications (ASMs), with special reference to clobazam and valproate-interactions first characterized in dedicated pharmacokinetic studies (Geffrey et al., 2015; Gaston et al., 2017), further shaped by CBD’s markedly food-dependent oral absorption (Birnbaum et al., 2019). It also mentions clinically significant CYP3A-mediated interactions, such as the increased exposure to everolimus observed with CBD co-administration in tuberous sclerosis complex (Wray et al., 2023).

Dosing considerations are covered from initiation and titration to maintenance and maximum doses, together with the need for ADR monitoring. Finally, the author discusses gradual dose titration as a “cornerstone” of an effective and personalized therapy (Navarro).

In one of the preclinical studies of this special use, conducted by Fuentes-Mejía, the use of CBD alone or in combination with different anti-seizure medications (ASMs) was examined in the experimental model of drug-resistant epilepsy induced by intraperitoneal (i.p.) administration of 3- mercaptopropionic acid (3-MP). This model is characterized by the increasing brain expression of P-glycoprotein (P-gp)), refractory phenotype, seizure severity, and spontaneous death rate as seizure burden accumulates (Lazarowski et al., 2004; Enrique et al., 2017). Their results from Fuentes-Mejía et al. showed that administration of CBD without ASMs did not modify the mortality rate or the development of the drug-resistan henotype, suggesting that CBD alone has no anticonvulsant effect *per se*. Although, a meta-analysis of four randomized controlled trials in Dravet and Lennox-Gastaut syndromes found that CBD’s antiseizure efficacy over placebo was preserved irrespective of concomitant clobazam use. This report also emphasized that the magnitude of effect was numerically larger with clobazam co-therapy (Devinsky et al., 2020). According with these observations, in the Fuentes-Mejía study, the co-administration of CBD with phenobarbital or diazepam reduced not only the mortality rate but also the prevalence of drug-resistant phenotype, benefits that were not observed with valproic acid or lamotrigine. However, it is important to note that in this experimental study, the combining of CBD with oxcarbazepine increased the incidence of drug-resistant seizures. In the search for a mechanistic explanation of this phenomenon, authors explore, by computational docking, the interactions between CBD and sodium channel blockers at the NaV1.7 channel (a voltage-gated sodium channel encoded by the *SCN9A* gene). Authors suggests that the CBD binding to this channel, could alter the conformational NaV1.7 folding and may interfers with the inhibitory effects of oxcarbazepine (Fuentes-Mejia et al.). Because structural studies using cryo-electron microscopy (cryo-EM) and electrophysiological approaches have described state-dependent inhibition by CBD at two distinct binding sites on NaV channels; therefore, this mechanism could, at least in part, explain the antagonistic interaction between oxcarbazepine and CBD. However, this interpretation cannot be directly extrapolated to other sodium channel blockers, such as carbamazepine, eslicarbazepine, rufinamide and topiramate, as these compounds may interact with distinct binding sites. Therefore, functional confirmation of the proposed interaction should be specifically established for each drug combination. The other preclinical study by Cabral-Pereira et al., used the GASH/Sal hamster, a genetic model of audiogenic generalized tonic–clonic seizures whose epileptogenic focus is the inferior colliculus, to evaluate CBD (200 mg/kg, i.p.) in males and females, both acutely and after 14 days of twice-daily administration.

CBD applied prior to stimulation reduced audiogenic seizure severity in a time-dependent manner, with the greatest protection after chronic dosing (a median reduction of 5 points in the categorized seizure index at day 14 with respect to each animal’s own baseline), and prolonged latency to seizure onset in both sexes, earlier in females (4 h) than in males (day 7). Complete seizure suppression after chronic treatment was more frequent in females than in males (37.5% vs. 12.5%), although direct male–female comparisons did not reach statistical significance and should therefore be regarded as exploratory. They also documented that higher serum and brain CBD concentrations were associated with lower seizure severity. Chronic treatment was well tolerated, with no changes in body weight or liver function, although platelet counts increased in both sexes and white blood cell counts decreased in CBD-treated females. It is worth noting that, at inferior colliculus level, a sex-dependent differences were observed in serotonergic, endocannabinoid, purinergic, and *Sigmar1*-related neuroprotective pathways; within this molecular context, seizure induction modulated *Trpv1* and *Slc29a1*, whereas chronic CBD produced pathway-specific, sex-dependent changes involving *5-Htr1a*, *Adora1* and *Cnr1*, without eliciting widespread transcriptional remodeling (Cabral-Pereira et al.). In this regard, the sigma-1 receptor (encoded by *Sigmar1*) has also been recognized as a potential therapeutic target for modulating seizure threshold and related comorbidities in seizure disorders (Vavers et al., 2023).

In fact, some aspects of the preclinical evidence presented here appears to be conflicting, since the benefit of CBD in the 3-MP model depended on its combination with selective ASMs, as mentioned in the Fuentes-Mejía study. Furthermore, a recent study reported that subchronic CBD administration after experimental induction of status epilepticus (SE) by pilocarpine, did not improve the seizure threshold during the epileptogenic silent period; strikingly, CBD worsened the hypersensitivity state and delayed recovery following SE, suggesting that orally administered CBD, at a human-equivalent therapeutic and safe dose, does not prevent the development of epileptogenesis process after SE (Gómez et al., 2026).

Taken together, these preclinical observations—showing that CBD’s benefit in models of pharmacoresistance depends critically on the co-administered ASM, and that it may even be detrimental in the post-status-epilepticus period—argue for caution rather than generalization. They reinforce the need for a more judicious, individualized use of CBD in combination with different ASMs, within the framework suggested by Navarro in the accompanying review.

Finally, the work presented by Pesántez Ríos et al. addresses the role of CBD as an anti-inflammatory agent which, acting as a multi-target drug, reduces the concentrations of pro-inflammatory cytokines, chemokines, reactive oxygen species (ROS), and neurotoxic factors released by microglia (Pesántez Ríos et al.). These properties are framed within the context of neuroinflammation modulation, proposed as a promising therapeutic strategy in drug-resistant epilepsies (Ramos et al., 2025). It is important to note that, to date, no direct link has been established between CBD-mediated reduction of inflammatory markers and clinical reduction of seizures; therefore, this relationship remains a hypothesis requiring specific clinical research, rather than a proven mechanism of direct anticonvulsant action of CBD.

Refractory status epilepticus (RSE), super-refractory status epilepticus (SRSE), new-onset refractory status epilepticus (NORSE) and febrile infection-related epilepsy syndrome (FIRES) are all severe convulsive entities characterized by their difficult pharmacological control, despite broad-spectrum therapeutic strategies. Also, they are a clinical emergency in which the uncontrolled seizures themselves can induce both cerebral and systemic inflammatory processes that worsens the drug-resistant epileptic condition (Janigro et al., 2013) and have a high risk of developing multiple organ failure (MOF) and finally the patients’ death.

The drug-resistant phenotype and highly complex inflammatory nature of RSE/SRS/NORSE/FIRE, not only perpetuate the convulsive state but also increase the death ratio, conditions resembling systemic inflammatory response syndrome (SIRS), as an initial phase of a complex inflammatory process, that carries a high-risk of MOF and patient death (Baddam and Burns, 2025). Interestingly, CBD treatment has been experimentally shown to decrease the levels of inflammatory cytokines in an experimental model of SIRS, suggesting that CBD might provide an opportunity to manage in these severe acute and chronic inflammatory disorders (Trivedi et al., 2022).

The systemic inflammatory state is a common condition in epileptic patients, and a long list of inflammation products or mediators have been suggested as biomarkers of seizures susceptibility while some anti-inflammatory drugs have been shown to reduce the seizures frequency of epileptic in both animals and humans (Vezzani et al., 2019; Stredny et al., 2023). Furthermore, elevated plasma concentrations of proinflammatory mediators have been documented in patients after SE. (Rejdak et al., 2023), and this massive release of cytokines can induce the overexpression of P-glycoprotein (P-gp) (Shi et al., 2025), triggering a ‘vicious cycle’ that increases the resistance to pharmacological treatment and susceptibility to further seizures (Janigro et al., 2013).

Several molecular mechanisms contributing to neuroinflammation in RSE are increasingly recognized, and evidence suggests that neuroinflammation plays a role in the initiation, propagation, and perpetuation of RSE, SRSE, NORSE, FIRES. An underlying inflammatory or autoimmune process is often suspected or identified in these syndromes (Samarasekera et al., 2025). Furthermore, prolonged uncontrolled seizures of RSE and NORSE are associated with elevated CSF and serum levels of interleukin-6 (IL-6) and tumor necrosis factor-alpha (TNF-α) (Vezzani et al., 2019). These findings suggest that CBD may have a significant anti-inflammatory effect in the most severe cases of SE, in accordance with the information reviewed in the work of Pesántez Ríos et al.

In this regard, under the presence of IL-1, IL-6 and TNFα, a functional overexpression of the P-glycoprotein (P-gp) resulting in a decreased Rho123 cellular accumulation was documented (Iqbal et al., 2012); *in vitro* experiments showed that CBD have a potent inhibitory effect on Rho123 transport, in cultures of both vascular endothelial cells and astrocytes, resulting in a high intracellular accumulation of Rho123, and this inhibitory property could allow greater access of ASMs to the CNS through the BBB (Auzmendi et al., 2020).

By curbing this inflammatory phenomenon and modify the AMSs pharmacokinetics (reduce the expression or blocking the function of P-gp and CYP-450), CBD breaks this vicious cycle, sensitizes refractory epilepsies to ASMs (Patel et al., 2021; Caraballo et al., 2022; Navarro; Feria-Romero et al., 2025). However, we must point out that the possible modulation by CBD of the inflammatory condition of RSE, SRSE, NORSE, and FIRES remains an open and speculative question, requiring specific experimental and clinical research protocols.

Additionally, these convulsive conditions can induce ferroptosis, a mechanism that is activated because of the high convulsive load (Mao et al., 2019), but which in turn, when brain iron overload and ferroptosis are activated by other causes, can also induce convulsions (Auzmendi and Lazarowski, 2025) and CBD could play a protective role (Kowalski et al., 2026).

We wish to be explicit about the limits of the current evidence base: human data on CBD in these emergency settings are confined to isolated case reports and small series, and current international consensus recommendations for NORSE and FIRES do not include CBD as a first- or second-line therapy. While the few initial cases of RSE treated with low or late doses of CBD did not yield positive results, there is also clinical evidence supporting the protective role of CBD, demonstrating effective seizure control and EEG normalization when higher doses (15–20 mg/kg/day) were used in combination with anti-seizures medications (ASMs) (Aydemir and Kandula, 2022). This is consistent with the findings of a recent meta-analysis in which 9 of 11 patients with RSE showed favorable results after treatment with CBD (Duda and Reinert, 2022).

It is well known that practical barriers further complicate any emergency application of CBD, due to its slow and variable absorption via the oral route, which depends on food intake, and no validated rapid-rescue preparation of clinical use currently exists, and factors typical of critical illness—enteral feeding interruption, hepatic dysfunction, and interactions with clobazam, valproate and anesthetic agents—could unpredictably alter its pharmacokinetics and safety profile in this population.

In this regard, several experimental studies have shown the viability of using different CBD formulations administered intravenously (Sánchez de Medina et al., 2023; Koch et al., 2024), and a preclinical trial in mice demonstrated that a single low intravenous bolus dose of 10 mg/kg of CBD dissolved in fat emulsion achieved similar therapeutic results to high oral doses of 100 mg/kg (Xu et al., 2019). Therefore this i.v. administration of CBD could be very useful in the critical cases mentioned above. All these reports, and the information mentioned above, indicate the need for the development of well-designed translational and clinical research—including studies of formulation, administration routes and their pharmacokinetics under critical-illness conditions—to define whether, and how, CBD might eventually have a protective role against the proinflammatory ‘cytokine storm’, and additionally to reverse the drug-resistant phenotype, helping in for the emergency management of RSE, SRSE, NORSE or FIRES, beyond drug-resistant epilepsies.

## References

[B1] Auzmendi J. Lazarowski A. (2025). Ferroptosis: is it both cause and effect in refractory epilepsy? Explor Neurosci. 4, 100692. 10.37349/en.202M00692

[B2] Auzmendi J. Palestro P. Blachman A. Gavernet L. Merelli A. Talevi A. (2020). Cannabidiol (CBD) inhibited rhodamine-123 efflux in cultured vascular endothelial cells and astrocytes under hypoxic conditions. Front. Behav. Neurosci. 14, 32. 10.3389/fnbeh.2020.00032 32256321 PMC7090129

[B3] Aydemir S. Kandula P. (2022). High dose cannabidiol (CBD) in the treatment of new-onset refractory status epilepticus (NORSE). Seizure 94, 126–128. 10.1016/j.seizure.2021.11.020 34896815

[B4] Baddam S. Burns B. (2025). “Systemic inflammatory response syndrome,” in Statpearls. Treasure Island (FL) (Treasure Island, FL: StatPearls Publishing). 31613449

[B5] Birnbaum A. K. Karanam A. Marino S. E. Barkley C. M. Remmel R. P. Roslawski M. (2019). Food effect on pharmacokinetics of cannabidiol oral capsules in adult patients with refractory epilepsy. Epilepsia 60 (8), 1586–1592. 10.1111/epi.16093 31247132

[B6] Caraballo R. Reyes G. Demirdjian G. Huaman M. Gutierrez R. (2022). Longterm use of cannabidiol-enriched medical cannabis in a prospective cohort of children with drug-resistant developmental and epileptic encephalopathy. Seizure 95, 56–63. 10.1016/j.seizure.2022.01.001 34999381

[B7] Devinsky O. Cross J. H. Laux L. Marsh E. Miller I. Cannabidiol in Dravet Syndrome Study Group (2017). Trial of cannabidiol for drug-resistant seizures in the Dravet syndrome. N. Engl. J. Med. 376 (21), 2011–2020. 10.1056/NEJMoa1611618 28538134

[B8] Devinsky O. Thiele E. A. Wright S. Checketts D. Morrison G. Dunayevich E. (2020). Cannabidiol efficacy independent of clobazam: meta-analysis of four randomized controlled trials. Acta Neurol. Scand. 142 (6), 531–540. 10.1111/ane.13305 32592183 PMC7689899

[B9] Duda J. Reinert J. P. (2022). Cannabidiol in refractory status epilepticus: a review of clinical experiences. Seizure 103, 115–119. 10.1016/j.seizure.2022.11.006 36399869

[B10] Enrique A. Goicoechea S. Castaño R. Taborda F. Rocha L. Orozco S. (2017). Bruno blanch New model of pharmacoresistant seizures induced by 3-mercaptopropionic acid in mice. Epilepsy Res. 129:8–16. 10.1016/j.eplepsyres.2016.10.012 27875747

[B11] Feria-Romero I. A. Rocha L. Reyes-Cuayahuitl A. Martínez-Juárez I. San-Juan D. Escamilla-Nuñez C. (2025). Analysis of six CYP450 genetic variants regarding the response to cannabidiol combined with anticonvulsant medication in mexican patients with drug-resistant epilepsy. Front. Pharmacol. 16, 1626054. 10.3389/fphar.2025.1626054 40918507 PMC12408562

[B12] Gaston T. E. Bebin E. M. Cutter G. R. Liu Y. Szaflarski J. P. Program (2017). Interactions between cannabidiol and commonly used antiepileptic drugs. Epilepsia 58 (9), 1586–1592. 10.1111/epi.13852 28782097

[B13] Geffrey A. L. Pollack S. F. Bruno P. L. Thiele E. A. (2015). Drug-drug interaction between clobazam and cannabidiol in children with refractory epilepsy. Epilepsia 56 (8), 1246–1251. 10.1111/epi.13060 26114620

[B14] Gómez C. T. Fernández F. Jara A. Borda N. Moscovicz F. Yauri-Huaman Y. (2026). Subchronic cannabidiol (CBD) treatment during the silent period fails to prevent increased seizure susceptibility following lithium-pilocarpine-induced status epilepticus. Brain Sci. 16 (8), 851. 10.3390/brainsci16080851 42651161 PMC13510346

[B15] Iqbal M. Ho H. L. Petropoulos S. Moisiadis V. G. Gibb W. Matthews S. G. (2012). Pro-inflammatory cytokine regulation of P-glycoprotein in the developing blood-brain barrier. PLoS One 7 (8), e43022. 10.1371/journal.pone.0043022 22973436 PMC3433182

[B16] Janigro D. Iffland P. H. Marchi N. Granata T. (2013). A role for inflammation in status epilepticus is revealed by a review of current therapeutic approaches. Epilepsia. 54 (06):30–32. 10.1111/epi.12271 24001067 PMC4061983

[B36] Koch N. Jennotte O. Lechanteur A. Deville M. Charlier C. Cardot J. M (2024). An intravenous pharmacokinetic study of cannabidiol solutions in piglets through the application of a validated ultra-high-pressure liquid chromatography coupled to tandem mass spectrometry method for the simultaneous quantification of CBD and Its carboxylated metabolite in plasma. Pharmaceutics. 16 (1), 140. 10.3390/pharmaceutics16010140 38276510 PMC10820640

[B34] Kowalski L. Colucci A. C. M. Dos Santos J. B. Vuaden B. W. Herlinger A. L. Hallak J. E. C. (2026). Differential effects of cannabidiol and cannabigerol on cognition, neuroinflammation, and blood-brain barrier integrity in a rat model of iron overload. Neurotox Res. 44 (5), 48. 10.1007/s12640-026-00826-x 42663810 PMC13524965

[B19] Kwan P. Arzimanoglou A. Berg A. T. Brodie M. J. Hauser W. A. Mathern G. (2010). Definition of drug resistant epilepsy: consensus proposal by the ad hoc task force of the ILAE commission on therapeutic strategies. Epilepsia 51 (6), 1069–1077. 10.1111/j.1528-1167.2009.02397.x 19889013

[B20] Lazarowski A. Ramos A. J. García-Rivello H. Brusco A. Girardi E. (2004). Neuronal and glial expression of the multidrug resistance gene product in an experimental epilepsy model. Cell Mol. Neurobiol. 24 (1), 77–85. 10.1023/b:cemn.0000012726.43842.d2 15049512 PMC11529958

[B21] Mao X. Y. Zhou H. H. Jin W. L. (2019). Ferroptosis induction in pentylenetetrazole kindling and pilocarpine-induced epileptic seizures in mice. Front. Neurosci. 13, 721. 10.3389/fnins.2019.00721 31379480 PMC6652743

[B22] Patel S. Grinspoon R. Fleming B. Skirvin L. A. Wade C. Wolper E. (2021). The long-term efficacy of cannabidiol in the treatment of refractory epilepsy. Epilepsia 62 (7), 1594–1603. 10.1111/epi.1693 34050682

[B23] Ramos A. J. Lazarowski A. Vega-García A. Buriticá-Ramírez E. Auzmendi J. Becerra-Hernández L. V. (2025). Modulation of neuroinflammation as a therapeutic strategy for the control of epilepsy. Seizure, 131:458 458–470. 10.1016/j.seizure.2025.08.023 40876369

[B24] Rejdak K. Pikulicka A. Piekarska M. Pacek K. Płachta K. (2023). Inflammation as treatment target for status epilepticus. Curr. Neuropharmacol. 21 (3), 708–714. 10.2174/1570159X21666221117152249 36397618 PMC10207918

[B25] Samarasekera S. Thilak S. Patel A. Nasa P. Ahmed Z. Jacques L. (2025). Refractory status epilepticus and therapeutic uncertainties: a comprehensive review on targeting neuroinflammation and rationale for developing a platform trial. Adv. Sci. (Weinh) 12 (47), e09668. 10.1002/advs.202509668 41017562 PMC12713006

[B35] Sánchez de Medina A. Serrano-Rodríguez J. M. Díez de Castro E. García-Valverde M. T. Saitua A. Becero M. (2023). Pharmacokinetics and oral bioavailability of cannabidiol in horses after intravenous and oral administration with oil and micellar formulations. Equine Vet J. 55 (6), 1094–1103. 10.1111/evj.13923 36624043

[B26] Shi J. Xie J. Li Z. He X. Wei P. Sander J. W. (2025). The role of neuroinflammation and network anomalies in drug-resistant epilepsy. Neurosci. Bull. 41 (5), 881–905. 10.1007/s12264-025-01348-w 39992353 PMC12014895

[B27] Stredny C. Rotenberg A. Leviton A. Loddenkemper T. (2023). Systemic inflammation as a biomarker of seizure propensity and a target for treatment to reduce seizure propensity. Epilepsia Open 8 (1), 221–234. 10.1002/epi4.12684 36524286 PMC9978091

[B28] Thiele E. A. Marsh E. D. French J. A. Mazurkiewicz-Beldzinska M. Benbadis S. R. GWPCARE4 Study Group (2018). Cannabidiol in patients with seizures associated with lennox-gastaut syndrome (GWPCARE4): a randomised, double-blind, placebo-controlled phase 3 trial. Lancet 391 (10125), 1085–1096. 10.1016/S0140-6736(18)30136-3 29395273

[B29] Thiele E. A. Bebin E. M. Bhathal H. Jansen F. E. Kotulska K. GWPCARE6 Study Group (2021). Add-on cannabidiol treatment for drug-resistant seizures in Tuberous sclerosis complex: a placebo-controlled randomized clinical trial. JAMA Neurol. 78 (3), 285–292. 10.1001/jamaneurol.2020.4607 33346789 PMC7754080

[B30] Trivedi M. K. Mondal S. Gangwar M. Jana S. (2022). Anti-inflammatory potential of cannabidiol (CBD) on combination of caecal slurry, LPS, and E. Coli-induced systemic inflammatory response syndrome (SIRS) in sprague dawley rats. Inflammopharmacology 30 (1), 225–232. 10.1007/s10787-021-00901-z 34997430

[B31] Vavers E. Zvejniece L. Dambrova M. (2023). Sigma-1 receptor and seizures. Pharmacol. Res. 191, 106771. 10.1016/j.phrs.2023.106771 37068533 PMC10176040

[B32] Vezzani A. Balosso S. Ravizza T. (2019). Neuroinflammatory pathways as treatment targets and biomarkers in epilepsy. Nat. Rev. Neurol. 15 (8), 459–472. 10.1038/s41582-019-0217-x 31263255

[B33] Wray L. Stott C. Jones N. Wright S. VanLandingham K. (2023). Pharmacokinetic drug-drug interaction with coadministration of cannabidiol and everolimus in a phase 1 healthy volunteer trial. Clin. Pharmacol. Drug Dev. 12 (8), 761–768. 10.1002/cpdd.1262 37132402

[B37] Xu C. Chang T. Du Y. Yu C. Tan X. Li X. (2019). Pharmacokinetics of oral and intravenous cannabidiol and its antidepressant-like effects in chronic mild stress mouse model. Environ Toxicol Pharmacol. 70, 103202. 10.1016/j.etap.2019.103202 31173966

